# Evaluation of the Interaction of Sex Hormones and Cardiovascular Function and Health

**DOI:** 10.1007/s11897-022-00555-0

**Published:** 2022-05-28

**Authors:** Myrthe M. A. Willemars, Miranda Nabben, Job A. J. Verdonschot, Martijn F. Hoes

**Affiliations:** 1 Department of Genetics & Cell Biology, Faculty of Health, Medicine and Life Sciences, Maastricht University, Maastricht, the Netherlands; 2 CARIM School for Cardiovascular Diseases, Maastricht, the Netherlands; 3 Department of Clinical Genetics, Maastricht University Medical Center+, Maastricht, the Netherlands; 4 Department of Cardiology, Faculty of Health, Medicine and Life Sciences, Maastricht University, Maastricht, the Netherlands

**Keywords:** Sex hormones, Cardiovascular disease, Heart failure, Hormone therapy

## Abstract

**Purpose of Review:**

Sex hormones drive development and function of reproductive organs or the development of secondary sex characteristics but their effects on the cardiovascular system are poorly understood. In this review, we identify the gaps in our understanding of the interaction between sex hormones and the cardiovascular system.

**Recent Findings:**

Studies are progressively elucidating molecular functions of sex hormones in specific cell types in parallel with the initiation of crucial large randomized controlled trials aimed at improving therapies for cardiovascular diseases (CVDs) associated with aberrant levels of sex hormones.

**Summary:**

In contrast with historical assumptions, we now understand that men and women show different symptoms and progression of CVDs. Abnormal levels of sex hormones pose an independent risk for CVD, which is apparent in conditions like Klinefelter syndrome, androgen insensitivity syndrome, and menopause. Moreover, sex hormone–based therapies remain understudied and may not be beneficial for cardiovascular health.

## Introduction

Cardiovascular diseases (CVDs) are the leading causes of death worldwide according to the World Health Organization [1]. Up to 32% of global deaths were due to CVDs in 2019, of which 85% were directly linked to stroke and myocardial infarction [1]. These numbers underscore the global burden of CVDs to health care systems and the economic consequences. The main risk factors of CVDs include hypertension, obesity, high fasting plasma glucose, and smoking [2]. While the number of people that smoke is decreasing, other risk factors are becoming more prevalent as the western (high-fat) diet and a sedentary lifestyle are spreading worldwide [3]. Regardless of environmental factors, it has also become apparent that men and women present distinctly different disease profiles [4, 5•]. Most risk factors for CVD affect women differently from men, which is also reflected in disease progression and CVD phenotypes [5•]. For example, systemic inflammation [5•, 6], vascular stiffness [7], and concentric left ventricular (LV) hypertrophy [8] are more prevalent in women. As such, women are more likely to develop heart failure (HF) with preserved ejection fraction (HFpEF) [9, 10] and lower quality of life [11], especially when combined with obesity and advanced age [12]. By contrast, atherosclerosis [13, 14] and ischemic heart disease [12] are more prevalent in men and often result in HF with reduced ejection fraction (HFrEF) [15], which is associated with higher mortality at a younger age compared to HFpEF [16, 17]. Therefore, women experience different symptoms and disease progression, and women also responded differently to heart failure medication like angiotensin-converting-enzyme (ACE) inhibitors and angiotensin-receptor blockers (ARBs) [18]. The current dogma states that sex differences are mostly derived from hormonal signaling that is associated with the presence of the specific sex chromosomes [19, 20]. However, X-linked gene dosage may be an additional mechanism that drives early embryonic processes toward a sex-specific molecular profile [21]. Indeed, the underlying mechanisms that determine medically relevant sex differences are complex and not fully understood. Notably, the incidence of CVDs rises significantly after a defined moment in life after which sex hormone levels drop radically and is commonly known as the menopause in women and late-onset hypogonadism, its male equivalent [22••, 23]. While these events are physiological, some diseases (e.g., polycystic ovarian syndrome [PCOS] or some types of cancer) may also affect sex hormone levels and could predispose to CVDs. In this review, we describe how abnormal levels of sex hormones and their downstream signaling can affect cardiovascular health and increase the risk for CVDs, and how hormone levels fluctuate in heart failure patients. We also summarize the results of clinical trials and how sex hormone therapy can alleviate CVD symptoms and improve health status.

## Production and Function of Sex Hormones

### Sex Hormone Steroidogenesis

Sex hormones are a class of hormones that initiate and control development and function of the reproductive organs or the development of secondary sex characteristics. The main sex hormones are estrogens, androgens, and progestogens. Estrogens are a subclass consisting of estrone (E1), 17β-estradiol (E2), estriol (E3), and estetrol (E4), of which 17β-estradiol is the most prominent [24, 25]. Progesterone (P4) is the main progestogen [26–28], whereas testosterone and its more potent derivative dihydrotestosterone (DHT) are considered to be the main androgens.

Sex hormones are synthesized from cholesterol by steroidogenesis [25]. Cholesterol is first converted into progestogens, followed by production of androgens and subsequently estrogens [25, 27]. The production of progesterone and androgens is facilitated by the cytochrome P450 (CYP) superfamily of enzymes and hydroxysteroid dehydrogenases (HSDs) [25]. The conversion of androgens to the estrogens estrone and estradiol is catalyzed by the special CYP enzyme aromatase, whereas the production of DHT is mediated by 5α-reductase [24, 27]. Steroidogenesis mainly takes place in gonadal tissues, after which further conversions can be facilitated by specific extragonadal tissues, such as adipose tissue, skeletal muscle, breast tissue, and prostate and liver [24, 27]. These enzymes are ubiquitous but are most active in the liver. Recently, both aromatase and 5α-reductase activity were shown in the heart [24, 29, 30, 31••]. Plasma levels of sex hormones are influenced by multiple factors, such as sex, age, phase of the menstrual cycle, and pregnancy status, which contribute to inter-person differences.

### Sex Hormone Receptors and Pathways

**Fig. 1:**
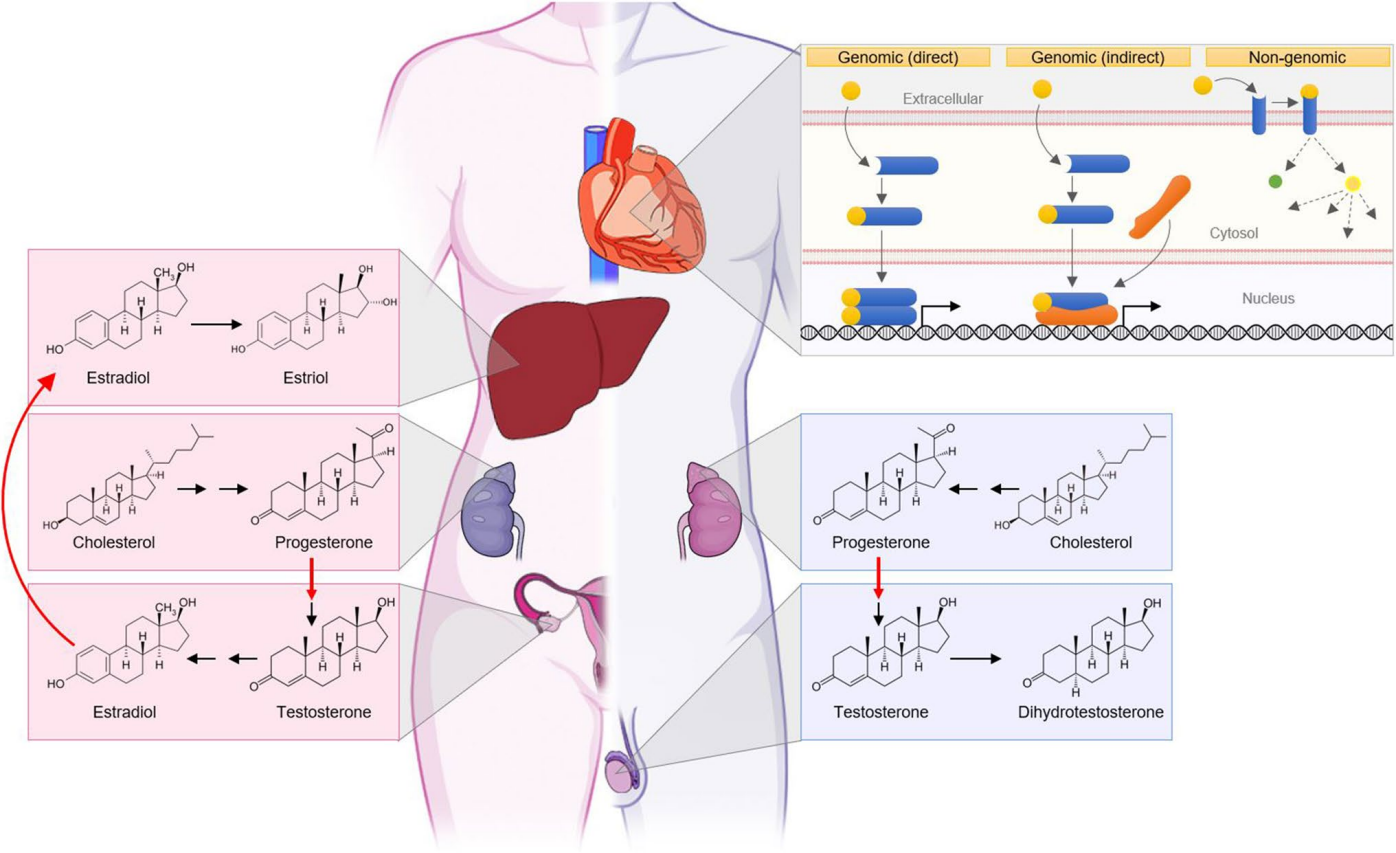
Schematic overview of sex hormone synthesis and cellular effects. In both men and women, cholesterol is the steroidogenic substrate for progesterone production in the adrenal gland. Besides being a functional hormone itself, progesterone is converted to testosterone in the ovaries of women (left) and in the testes of men (right). Subsequently, testosterone is converted to estradiol in the ovaries before being secreted into the circulation. Other organs—like the liver—produce estriol from estradiol. In men, testosterone is also converted to the more potent dihydrotestosterone in the testes. Once sex hormones (yellow) reach the target cells in the heart, they may induce downstream targets by direct or indirect genomic pathways via gene expression, or by interacting with a membrane-bound receptor and other effector proteins in various organelles

Sex hormones exert their effects by binding to their designated receptors located in target tissues. The estrogen receptors alpha (ERα) and beta (ERβ) are present in the cytoplasm or incorporated into the plasma membrane and translocate to the nucleus upon stimulation, while G protein–coupled estrogen receptor 1 (GPER1; previously known as GPR30) is exclusive to the plasma membrane [24, 25, 31••]. The progesterone receptors A (PR-A) and B (PR-B) are located in the cytoplasm and can translocate to the nucleus. A subclass of progesterone receptors (mPR-α, mPR-β, mPR-γ, mPR-δ, and mPR-ε) remains bound to the plasma membrane upon activation and functions in a GABA receptor–dependent fashion [32, 33]. Like ERα and ERβ, the androgen receptor (AR) resides in the cytoplasm in its inactive state [26, 31••]. Receptor-ligand interaction activates genomic or non-genomic signaling mechanisms. For the genomic pathway, receptors dimerize upon stimulation and translocate to the nucleus where they either directly or indirectly, via other transcription factors, bind to their DNA response elements and alter gene expression. Additionally, stimulated receptors can activate signal transduction pathways to induce more rapid responses [25, 31••, 33]. The ultimate effect primarily depends on the type of sex hormone, activated receptor, and cell type. Sex hormones can affect cellular function in almost all human cell types, but their specific downstream effects vary per cell type due to epigenetic differences among target cells. An overview of sex hormone production in men and women and downstream pathway activation is illustrated in Fig. 1.

### Molecular Effects of Sex Hormones on the Cardiovascular System

Sex hormones have a myriad of effects due to various combinations of dimerization with other (nuclear) receptors and transcription factors. Receptor presence and combinations differ per cell type, which orchestrates downstream effects of each sex hormone. We have summarized demonstrated molecular effects of sex hormones in the cardiovascular system in this section and in Table 1.

**Table 1:** Sex hormones and their effects on cells of the cardiovascular system

Sex hormone	Cell type	Effect	References
17β-estradiol	Cardiomyocytes	↓ Hypertrophy	[34–37]
		↑ ATP synthesis	[38]
		↓ ROS production	[39]
		↓ Contractility	[40]
		↓ Apoptosis	[41, 42, 55]
	Endothelial cells	↑ Vasorelaxation	[44–49]
		↑ Proliferation	[45, 46, 48, 49]
		↑ Migration	[45, 46, 48, 49]
		↑ Angiogenesis	[50•]
	Vascular smooth muscle cells	↑ Vasorelaxation	[51]
		↓ Proliferation	[37, 52]
		↓ Migration	[37, 52]
		↓ Inflammation	[53]
	Fibroblasts	↓ Fibrosis	[35, 54, 55]
Testosterone	Cardiomyocytes	↑ Hypertrophy	[56–58]
		↑ Contractility	[59]
		↑ ROS production	[61]
		↑ Glucose uptake	[60]
	Endothelial cells	↑ Vasorelaxation	[62–64]
		↑ Proliferation	[73]
	Vascular smooth muscle cells	↑ Vasorelaxation	[65–72]
		↑ / ↓ Inflammation	[74–77]
		↑ Apoptosis	[78]
Progesterone	Cardiomyocytes	↑ β-oxidation	[79]
		↑ Proliferation	[80]
	Endothelial cells	↑ Vasorelaxation	[81–83]
		↓ Atherogenesis	[84]
		↑ ROS production	[85]
	Vascular smooth muscle cells	↑ Vasorelaxation	[81–83]
		↓ Atherogenesis	[84]
		↑ ROS production	[85]

#### ***Estrogens***

Estrogens have various effects on the cardiovascular system. In human cardiomyocytes, estrogens exert anti-hypertrophic effects mediated by myocyte-enriched calcineurin-interacting protein (MCIP-1), Histone deacetylases (HDACs), and natriuretic peptide precursor A (NPPA) [34–37]. Additionally, they improve mitochondrial efficiency, by increasing ATP synthesis while lowering ROS production via increased superoxide dismutase (SOD) activity [38, 39]. Also, cardiac electrical conductance can be affected, as estrogens decrease the expression and activity of ion channels, thereby lowering contractility [24, 36, 40]. Furthermore, estrogens exert various anti-apoptotic effects on cardiomyocytes, including activation of the PI3K/Akt and SIRT1 pathways and increasing miR-22 [41–43].

With regard to the vascular system, estrogens promote vasorelaxation, proliferation, and migration in endothelial cells (ECs) by increasing NO synthesis and expression of vasodilating factors, while lowering vasoconstrictive factors [32, 44–49]. Moreover, angiogenesis is stimulated by 17β-estradiol via increased expression of vascular endothelial growth factor (VEGF), which subsequently increases endothelial nitric oxide synthase (eNOS) activity [50•]. Vasorelaxation of vascular smooth muscle cells (VSMCs) is similarly stimulated via NO, followed by alteration of ion channel activity [51]. Furthermore, estrogens stimulate factors that ultimately inhibit EC proliferation and migration [37, 52]. Combined with inhibition of the same processes in VSMCs, estrogens promote regeneration while preventing atherogenesis. Estrogens can reduce inflammation by inhibiting activation of NF-kB and associated expression of pro-inflammatory cytokines [53]. Fibrosis is also attenuated by inhibiting transforming growth factor-beta (TGF-β), c-Jun N-terminal kinases (JNK), matrix metalloproteinase 2 (MMP2), and certain cell cycle proteins in fibroblasts [35, 54, 55].

#### ***Androgens***

In contrast to estrogens, the effects of androgens on the cardiovascular system remain relatively understudied with more controversial results. In cardiomyocytes, androgens exert pro-hypertrophic effects by stimulating various pathway axes related to protein synthesis, such as the Ca^2+^/calmodulin-dependent protein kinase (CaMKII), calcineurin/nuclear factor of activated T-cells (NFAT)/glycogen synthase kinase-3 beta (GSK-3β), and the mammalian target of rapamycin complex 1 (mTORC1)/S6 Kinase 1 (S6K1) axes [56–58]. Additionally, testosterone stimulation enhances contractile function, via increased expression of β1-adrenergic receptors and ion channels (L-type Ca2 + channels, Na + /Ca2 + exchangers), thereby altering cardiac calcium handling and increasing contractile force [59]. Furthermore, testosterone may influence cardiac metabolism by upregulating the expression of pro-oxidant enzymes, such as NADPH oxidase, Xanthine oxidase, and cyclo-oxygenase 2 (COX-2), and by increasing Glucose transporter type 4 (GLUT4)–mediated glucose uptake [60, 61].

Vascular functioning was also shown to be affected by androgens as well; studies demonstrated vasorelaxation in both ECs and VSMCs [62–64]. Here, testosterone was shown to reduce vasoconstricting factors and increase NO production in ECs [62–64]. Subsequently, this NO produced in ECs can influence VSMCs, by stimulating the production of cGMP and cAMP, which eventually alters ion channel activity and causes vasorelaxation [65–72]. Furthermore, androgens stimulate EC proliferation via VEGF and cyclin proteins [73]. Regarding inflammation, more controversial results have been found. Both pro-inflammatory effects, by increased TNFα activity and expression of vascular adhesion molecules in ECs, as well as anti-inflammatory effects, via decreased adhesion factors, were found [74–77]. Moreover, apoptosis in VSMCs is increased via the extrinsic apoptotic pathway [78].

#### ***Progestogens***

The effects of progestogens in the cardiovascular system have not been studied extensively and further research is required to elucidate precise effects in this context. Thus far, progesterone was shown to increase β-oxidation and proliferation in cardiomyocytes [79, 80]. Moreover, stimulation of vasorelaxation of ECs and VSMCs were found, through increased eNOS activity and altered calcium availability via increased sarco/endoplasmic reticulum Ca^2+^-ATPase (SERCA) expression and activity [81–83]. Additionally, HDL and LDL seem to increase and decrease, respectively, after progesterone treatment, thereby reducing atherogenesis [84]. Progesterone is also found to increase NADPH oxidase activity in the vascular system, resulting in increased ROS production [85].

## Abnormal Levels of Sex Hormones Are Associated with Cardiovascular Disease

Sex hormone levels are tightly regulated through various mechanisms and are crucial for optimal health. Abnormal sex hormone levels (e.g., too high or too low) can cause a myriad of disruptive responses like specific comorbidities and increased risks for diseases. Hormone levels may fluctuate due to exogenous (or environmental) causes like malnutrition, stress, or inadequate use of supplements. If addressed in early stages, these atypical sex hormone levels can be reverted to normal, and the derived effects can be transient. Alternatively, sex hormone levels could be altered due to an endogenous driver like a chromosomal or genetic defect. Known endogenous causes for sex hormone disorders can be divided into three classes: disorders of sex development (I), inherited or acquired hypogonadism (II), and fertility disorders (III). These causes often result in abnormal levels of one or multiple sex hormones and have been associated with an increased risk of cardiovascular disease. Here, we provide a summary of disorders that are associated with aberrant sex hormone levels. Androgen insensitivity syndrome (AIS) is an X-linked genetic disorder in which individuals with an XY karyotype develop as females due to partial or complete lack of downstream signaling induced by androgens. Patients with AIS have increased body fat, high serum cholesterol, and impaired insulin sensitivity which resembles aspects of the metabolic syndrome and cardiovascular risk management is necessary [86]. Klinefelter syndrome (KS; 47,XXY karyotype) is the most frequent chromosome disorder causing male infertility and hypogonadism, and is associated with markedly reduced production of androgens (i.e., testosterone) [87•]. Patients with KS are at increased risk for cardiovascular mortality due to a combination of comorbidities. A recent meta-analysis shows that hormone replacement therapy with testosterone has beneficial effects on the metabolic profile of patients with KS, possibly lowering their cardiovascular risk [88]. PCOS is the most common endocrine disorder in women of reproductive age [89]. In contrast to the other described disorders, there is elevated androgen activity in women with PCOS. PCOS is a heterogeneous disease that can result in several phenotypes, and it has been suggested that the subgroup of patients with hyperandrogenism has the highest cardiovascular risk [90]. A meta-analysis demonstrated that women with PCOS have a twofold risk of arterial disease compared to women without PCOS, which was not due to a higher body mass index [91]. In addition to sex hormone disorders, the menopause in women has been described as a time of accelerated CVD risk [92]. The menopause signifies the permanent cessation of ovarian function and a transition to a nonreproductive phase of life which is associated with remarkable changes in hormonal patterns. Of note, not only can menopause increase the risk to CVD, therapies that aim to alleviate menopause-related symptoms, like hormone therapies, can also be deleterious for cardiovascular health.

### Sex Hormones as a Treatment Modality

Abnormal levels of sex hormones can be corrected through tailored therapies, which are mostly started to treat KS and menopause-related symptoms. In addition, an individual may undergo hormone replacement therapy (HRT) to align their secondary sexual characteristics with their gender identity. HRT is either given as feminizing hormone therapy (i.e., consisting of estrogens and antiandrogens) or as masculinizing therapy (i.e., consisting of androgens). Both forms of HRT are generally considered to be safe [93]. However, fundamental changes in hormone-driven homeostasis have been associated with the occurrence of several diseases, including reduced bone mineral density, coagulative complications, cancer, metabolic disorders, and CVD [94]. These associations remain to be confirmed as studies of cardiovascular complications of HRT are limited in number and large longitudinal epidemiological studies do not exist. Moreover, HRT presents broad and fundamental changes to a biological system. Conditions like KS and menopause have a specific etiology that can be targeted directly, and often require administration of a single hormone. Consequently, hormone therapies to treat these conditions have been studied more thoroughly. This section summarizes studies that were aimed at correcting abnormal hormone levels to prevent CVD entirely (i.e., primary prevention) or to limit disease progression when the first signs of CVD are noted (i.e., secondary prevention).

Testosterone replacement therapy is indicated in young men with primary sex hormone disorders like KS. In the past decades, testosterone is commonly prescribed to older men with low serum testosterone related to advanced age or obesity [95]. However, the effect of testosterone therapy on cardiovascular risk gives conflicting results, as extensively reviewed by Gagliano-Jucá and Basaria [96•]. Most evidence is gathered from population studies, retrospective studies, and randomized clinical trials. Population studies suggest an association between low serum testosterone levels and an increased risk of cardiovascular events [97, 98]. Some retrospective studies have shown higher risks of cardiovascular events in men receiving exogenous testosterone [99, 100]. Meta-analyses of randomized clinical trials investigating the effects of exogenous testosterone therapy report conflicting results [101, 102]. The main caveats in the published clinical trials are the lack of power and the short duration of follow-up to assess cardiovascular events. The TRAVERSE trial (NCT03518034) will be the first randomized controlled trial that is adequately powered to evaluate the incidence of cardiovascular events with administration of exogenous testosterone. The trial started in 2018 and will include 6,000 men between the age of 45 and 80 years old at high cardiovascular risk and a low serum testosterone (< 300 ng/dl) to receive either testosterone or placebo. The treatment and inclusion time per patient will be 60 months. It is therefore expected that it will take about 10 years before the final study results will be available. Until then, there are no evident cardiovascular benefits of testosterone therapy in men with low serum testosterone and therapy should be evaluated per individual.

The incidence of CVD increases in postmenopausal women due to a higher testosterone/estradiol ratio. Multiple high-quality studies have investigated the effect of hormone therapy (MHT) in postmenopausal women since the 1990s. The rational of menopausal MHT is that reversing the hormonal changes will reduce the cardiovascular risk and reduce morbidity and mortality. Two important aspects should be considered in determining benefits of MHT: timing of treatment (i.e., when to start treatment after onset of menopause) and the distinction between primary and secondary prevention of CVDs (i.e., the presence or absence of a cardiovascular medical history, respectively). There is large variety in the type of study (e.g., observational, case–control, clinical trials) and the used inclusion and exclusion criteria among these studies. A Cochrane systematic review by Boardman et al. gathered all information from clinical trials and investigated the effects of MHT on the prevention of CVD in menopausal women [103]; results have been summarized in Table 2.

**Table 2:** Summary of clinical trials and the benefits of MHT regarding primary and secondary prevention of CVD

	Primary	Secondary	Time since menopause onset	
	prevention	prevention	< 10 years	> 10 years
All-cause death	≈	≈	✓	≈
CV death	≈	≈	✓	≈
Stroke	✗	≈	≈	✗
Venous thromboembolism	✗	✗	✗	✗
Coronary heart disease	≈	≈	✓	≈

✓: *beneficial effects*

≈: *neutral outcome*

✗: *detrimental effects*

Early randomized controlled trials investigated the effect of secondary prevention for coronary heart disease (CHD[HERS trial]) and stroke (WEST trial) [104, 105]. Neither of the trials found a benefit of MHT on secondary prevention of the corresponding CVD. The HERS trial used a combination therapy of estradiol and progesterone in contrast to the WEST trial which only prescribed estrogen. The included women were postmenopausal on average for 18 and 25 years, respectively, before initiation of MHT. Trials investigating the role of MHT as secondary prevention are lacking in postmenopausal women within 10 years after onset of menopause. The Estrogen in Venous ThromboEmbolism Trial (EVTET) treated postmenopausal women with an average age of 56 years who had a history of a previous venous thromboembolism with estrogen and progesterone [106]. The study was terminated prematurely due to circumstantial evidence that MHT increased the risk of recurrent thromboembolism. A specific meta-analysis of randomized trials also stated that MHT as secondary prevention doubled the risk of venous thromboembolism in postmenopausal women (RR, 2.02[95% CI, 1.13–3.62])[103]. Furthermore, the Women’s Health Initiative (WHI) trials are the largest conducted trials investigating MHT as primary prevention for CVD in postmenopausal women [107–109]. The WHI I trial included postmenopausal women which were < 10 years in menopause and treated with a combination of estrogen and progesterone. The WHI II trial included women who were postmenopausal and had a hysterectomy and were treated with only estrogen. Overall, both trials did not find an effect of MHT on the prevention on coronary heart disease. However, in line with earlier observational studies, there was a non-significant trend that postmenopausal women between the age of 50 and 59 who had hot flashes benefitted from MHT [107–109]. Both studies noted an increased risk for stroke and venous thromboembolism, except in the subgroup of women between 50 and 59 years of age. Additionally, the Danish Osteoporosis Prevention Study (DOPS) tested oral estrogen alone in women with hysterectomy or combined with progesterone in postmenopausal women with an intact uterus [110]. Therapy initiation was done as soon as possible, reflected by a mean age of 50 years old in this study. The primary endpoint was a composite of heart failure, myocardial infarction, or death. After 10 years of treatment, the risk of the composite endpoint was half compared to untreated women (HR, 0.48[95% CI, 0.26–0.87]), and the prevalence of stroke was similar. Meta-analyses corroborated these results, with benefits regarding all-cause and cardiovascular mortality, a neutral effect on stroke, but a significant increased risk for venous thromboembolisms in women initiated on MHT < 10 years after menopause [103]. Overall, the use of MHT in menopausal women for either primary or secondary prevention has little if any benefit at all but increases the risk of stroke and venous thromboembolism significantly [107–109]. The only subgroup that might benefit from treatment are postmenopausal women who are younger than 60 years old and if MHT is initiated within 10 years after menopause onset [109]. A selection of clinical trials has been summarized in Table 3 to illustrate the diversity, complexity, and magnitude of trials on this topic.

**Table 3:** Overview of selected randomized controlled trials to study menopausal hormone therapy

Acronym	Country	Follow-up (y)	Patients	Therapy	Control	Outcome	Inclusion criteria	Mean age	Prevention	Time since menopause
HERS 1	US	4.1	2763	Conjugated equine estrogens + medroxyprogesterone acetate	Placebo	CHD	Postmenopausal with an intact uterus and CHD	67	Secondary	> 10 years
EVTET	Norway	1.3	140	Estradiol + norethisterone acetate	Placebo	Recurrent DVT	Previous venous TE	56	Secondary	< 10 years
WEST	US	2.8	664	17-β-estradiol	Placebo	Stroke or death	Postmenopausal women with recent ischemic stroke	72	Secondary	> 10 years
EPAT	US	2	222	17-β-estradiol	Placebo	Intima-media thickness of carotid	Postmenopausal women > 45 years without CVD and increased LDL	62	Primary	> 10 years
WHI I	US	5.6	16,608	Conjugated equine estrogens + medroxyprogesterone acetate	Placebo	CHD	Postmenopausal	63	Primary	< 10 years
WHI II	US	7.1	10,739	Conjugated equine estrogens	Placebo	CHD	Women with hysterectomy	64	Primary	< 10 years
WISDOM	International	1	4385	Conjugated equine estrogens + medroxyprogesterone acetate	Conjugated equine estrogens	Major CV event	Postmenopausal	63	Primary	> 10 years
DOPS	Denmark	10.1	1006	17-β-estradiol	No treatment	Major CV event	Recent postmenopausal	50	Primary	< 10 years

### Sex Hormone Changes in Patients with Heart Failure

HF may arise from consistent hormonal abnormalities and consequently worsening CVDs. Conversely, sex hormone levels may also be affected in patients with HF. Sex hormones are tightly regulated through complex feedback loops that may be disrupted by HF. Various groups have set out to determine levels of sex hormones in various populations of HF patients. In 2000, Moriyama et al. demonstrated an age-dependent decrease of the androgen Dehydroepiandrosterone sulfate (DHEA-S) in healthy male controls, but constitutively low DHEA-S levels in patients with HF, as well as a correlation between low DHEA-S levels and high NYHA class [111]. Later, Kontoleon et al. compared various sex hormone concentrations in patients with dilated cardiomyopathy to healthy male controls and found decreased levels of free testosterone, while DHEA-S remained unchanged [112]. However, no statistical analysis of sex hormone levels and HF severity was reported. Free testosterone was independently associated with HF mortality [113]. Similar studies demonstrated that testosterone, free testosterone, and DHEA-S were decreased in a cohort of male patients with systolic HF [114]. Additionally, an excess of sex hormone–binding globulin (SHBG) was detected in 38% of patients [114–116]. Univariate regression analysis indicated that deranged levels of (free) testosterone, DHEA-S, and SHBG were associated with HF severity [115]. Moreover, low serum estradiol levels in male chronic HFrEF patients were associated with increased serum levels of total testosterone, decreased DHEA-S, and a higher NYHA class [116]. Regarding HFrEF, higher estradiol and DHEA were associated with reduced risk (HR 0.60 and 0.59, respectively), while higher total testosterone/estradiol ratio associated with increased risk (HR 1.65) in the same study [117]. An extensive prospective study in the ARIC cohort compared sex hormone levels in male and female patients with HF to healthy controls and demonstrated lower levels of total testosterone in male patients, and lower SHBG in female patients [118]. DHEA-S was reduced in all patients with HF. In addition, decreased testosterone and DHEA-S associated with increased HF risk in men (HR 1.10 and 1.07, respectively) and decreased DHEA-S with increased HF(pEF) risk in women (HR 1.17 for all HF, HR 1.12 for HFpEF) [118].

Taken together, multiple studies have demonstrated that sex hormone levels are abnormal in patients with HF. Notably, most studies were limited to testosterone, DHEA-S, and SHBG and mostly included male patients. Recent studies have started including female patients and female sex hormones, but these studies are scarce. In line with this review, we expect to see more studies concerning female patients and female sex hormones in the near future. It should also be noted that these studies do not provide any evidence for a causal link between HF and sex hormone imbalance. The previous section has indicated how perturbed sex hormones may lead to CVD and HF, which may be a confounder for more severe HF. However, most clinical studies were observational and could not link sex hormone levels during HF to levels prior to HF onset. Ultimately, this might suggest that HF patients could be grouped based on sex hormone abnormalities and correcting the relevant hormone imbalance could be considered as a therapeutic approach for secondary (or even tertiary) HF prevention.

### Sex Differences in Genetic Cardiomyopathies

Sex hormones are essential for cardiovascular function secondary to sex development. CVDs are not exclusive to a single sex, but they do manifest in sex-dependent ways. For example, sex differences are well-described in non-ischemic forms of heart failure [5•, 119]. Strikingly, the same genetic variation is often more pathological in males than in females regardless of environmental factors [5•]. Most forms of cardiomyopathies have a strong genetic basis, and genetic screening is a first-tier test in the diagnostic work-up of patients with a new-onset cardiomyopathy [120••]. Although a pathological gene variant may be present, not all carriers will develop a cardiomyopathy. Such incomplete penetrance of genetic cardiomyopathies remains an important issue in clinical practice: which (genetic or non-genetic) factors determine the disease onset in gene carriers? One observation is consistent in all published studies: the penetrance is higher in males compared with females; the DCM population with a genetic variant is 60–80% male (varying due to specific gene variants) [119]. Although the sex-specific trend in penetrance is confirmed for almost every specific gene (e.g., *MYH7*, *LMNA*), how sex interacts with the penetrance of genetic cardiomyopathies is unknown. Leading hypotheses on this include (I) differences in exposure to triggers and predisposing cardiovascular risk profile (e.g., pregnancy or cardiotoxic chemotherapy in a predisposed person) and (II) the influence of sex hormones on (increased) cardiovascular risk [5•, 121, 122]. The latter hypothesis is often used to explain the differences in penetrance, although there are no studies that investigated the influence of specific sex hormones on disease penetrance in gene carriers. There is also no information on how menopause-related sex hormone fluctuations affect cardiomyopathy development in female gene carriers. The incomplete understanding of sex differences and the influence of sex hormones in disease penetrance of pathogenic gene variant carriers is an important knowledge gap and could be the first step towards a more personalized cardiac screening and treatment regime for male and female gene carriers.

## Conclusions

Historically, CVD was thought to be a condition that mostly affected men. However, it is now accepted that women are also susceptible to these diseases, albeit often with different symptoms and disease progression. We now know that men and women are physiologically markedly different and that understanding which mechanisms drive these differences is the key to developing better personalized approaches at various levels of health care. Sex hormones are the primary factors of interest in the search for sex-dependent mechanisms. The main gaps in our understanding range from very basic (e.g., identifying all sex hormones relevant to HF) to fundamental concepts (e.g., downstream targets and diverse cellular functions). Sex hormones have profound effects in development, homeostasis, and disease protection. However, their function is likely much broader than what is currently understood, which is confirmed by the observations of different hormone therapies utilized to rectify abnormal sex hormone levels as seen in, for example, AIS or menopause. We roughly understand the etiologies of sex hormone–related conditions because these are associated with excessive or impaired signaling of specific sex hormones. However, directed hormone replacement or inhibitor therapies have proven to be insufficient or even detrimental for general health. Cardiovascular parameters are among the principal endpoints of most hormone therapy-related studies. Specifically, MHT is the best studied therapy, but results are often inconclusive or contradictory. This is likely due to the complexity of menopause with the association of multiple hormones, varying ages of menopause onset, and diverse timing options to start therapy. In contrast, estrogen- or androgen-related therapies have been understudied and large randomized controlled trials pertaining cardiovascular outcomes have been initiated only recently. Such trials will be crucial to improve our understanding of sex hormone–related CVDs. Improving our understanding of the interaction between sex hormones and the cardiovascular system will advance treatment strategies, but also our understanding of sex-related risk factors and screening approaches.
